# Efficacy and safety of LongShengZhi capsule on functional recovery after acute ischemic stroke (LONGAN): Protocol and statistical analysis plan for a randomized, double-blind, placebo-controlled trial

**DOI:** 10.3389/fphar.2022.916421

**Published:** 2022-08-24

**Authors:** Dandan Zhang, Tingting Li, Anxin Wang, Luda Feng, Xinxing Lai, Kegang Cao, Li Zhou, Baolin Yang, Fangyuan Cui, Qingbin Li, Jinjuan Dou, Baoyun Qi, Chi Zhang, Ying Gao

**Affiliations:** ^1^ Dongzhimen Hospital, Beijing University of Chinese Medicine, Beijing, China; ^2^ Institute for Brain Disorders, Beijing University of Chinese Medicine, Beijing, China; ^3^ Department of Neurology, Beijing Tiantan Hospital, Capital Medical University, Beijing, China; ^4^ China National Clinical Research Center for Neurological Diseases, Beijing Tiantan Hospital, Capital Medical University, Beijing, China

**Keywords:** acute ischemic stroke, herbal medicine, randomized controlled trial, protocol, statistical analysis plan

## Abstract

**Background:** Due to limited time windows and technical requirements, only a small percentage of patients can receive reperfusion therapy for acute ischemic stroke (AIS). Previous studies have shown that LongShengZhi (LSZ) capsule can improve neurological outcomes in patients after AIS, yet those results have not been finally verified through rigorous randomized controlled trials. Thus, this trial was designed to further clarify the efficacy and safety of LSZ capsule for patients with AIS.

**Methods:** LSZ capsule on Functional Recovery after Acute Ischemic Stroke (LONGAN) trial is a prospective, multicenter, randomized, placebo-controlled, double-blind, parallel-group, superiority trial that enrolls patients from stroke and rehabilitation units in China. We will enroll 1,376 patients aged 18 years or older with AIS within 7 days of symptom onset and a National Institute of Health Stroke Scale (NIHSS) score of 4–15. Eligible patients will be randomized to receive either 2 g LSZ capsules three times a day or placebo LSZ capsules for 90 days. The primary outcome is the proportion of patients with favorable outcomes, as measured by the modified Rankin Scale (mRS) 90 days after randomization. The main safety outcome is the proportion of severe adverse events.

**Conclusion:** This study will be the first randomized, double-blind trial to evaluate the efficacy and safety of LSZ capsule in patients with AIS. In order to improve the transparency and reproducibility of the trial, the data will be analyzed in accordance with this pre-specified plan for statistical analysis to reduce bias due to selective analysis and reporting. This trial aims to provide high-quality evidence for the efficacy and safety of LSZ capsule for AIS.

## 1 Introduction

Stroke is a common disease affecting a quarter of the world’s population and the leading cause of death and disability (Feigin et al., 2018). Ischemic stroke (IS), accounting for 70% of strokes, is the most prevalent type of stroke and occurs mainly due to decreased cerebral blood flow caused by thrombosis or embolism (Wang et al., 2017). Current treatments for acute ischemic stroke (AIS) include reperfusion and antiplatelet therapy, as well as admission to the stroke unit. Reperfusion treatment, including intravenous thrombolysis and mechanical thrombectomy, resulted in good prognoses for about 30%–46% of patients (Goyal et al., 2016; Saver et al., 2016; Thomalla et al., 2020). Recombinant tissue plasminogen activator (r-tPA) is an approved primary treatment for AIS. Unfortunately, only a small proportion of patients can undergo reperfusion treatment due to the limited time window and technical requirements. Improvement of the neurological function of patients with AIS remains the main focus and is a difficult issue in clinical practice (Chamorro et al., 2016). During the past 2 decades, many neuroprotective agents have shown promising results in animal experiments, but whether they can be translated to clinical trials is still uncertain (Gladstone et al., 2002; O’Collins et al., 2006; Xu J. et al., 2021). To date, there are no guideline-approved pharmacotherapies for neuroprotection after AIS. Promotion of nerve function recovery remains one of the most important therapeutic strategies for AIS; thus, more appropriate and effective medication options are urgently needed for the improvement of neurological function after AIS.

Chinese medicine (CM), a unique and complicated medical system, has been developed over thousands of years and is widely applied as a supplementary and complementary therapy to Western medicine in IS treatment. Some studies have already provided evidence of potential anti-inflammatory and neuroprotective effects of natural medicines (Xiong et al., 2018; Yang et al., 2020). The LongShengZhi (LSZ) capsule, a well-known traditional Chinese herbal medicine product for treating IS (Chang et al., 2016), is formulated through the modification of a representative prescription (Buyang Huanwu decoction) proposed by Wang Qing-ren during the Qing dynasty. The LSZ capsule was manufactured by Buchang Pharmaceutical Co., Ltd. (Shaanxi, China) and certified as compliant with good manufacturing practices and approved for the treatment of IS in 2001 by the Sino Food and Drug Administration. The LSZ capsule contains 12 natural medicines and 19 main active ingredients (see Supplementary Material). Many of the bioactive ingredients have neuroprotective effects; for example, paeoniflorin, one of the main components, has been proven to suppress oxidative stress and protect neurons (Zhong et al., 2015). Moreover, other studies have indicated that the LSZ capsule has anti-inflammatory and neuroprotective effects and can reduce the activation of platelets and endothelial cells (Li et al., 2019; Yang et al., 2020). Network pharmacology can be used to predict and identify the potential active component of herbal medicine (Xu et al., 2021; Zhang et al., 2022). Based on a network pharmacology analysis, the LSZ capsule can regulate the pathological process of IS through the modulation of inflammation processes, trophic factor secretion, and immune-related lymphocyte regulation (Sun et al., 2019). Also, the main active ingredients of ″Persicae Semen - Carthami Flos - Chuanxiong Rhizoma″ drug group could form a better docking mode and high affinity with extracellular signal - regulated protein kinase 2 (ERK2) and Janus kinase 2 (JNK2), which had a good activity of treating stroke (Xu et al., 2019a; Xu et al., 2022). Previous clinical reports have demonstrated that the LSZ capsule can prevent atherosclerosis and improve the prognosis and nerve function in IS (Liu, 2014). Additionally, it has been reported that LSZ can reduce Alzheimer’s disease (AD) Aβ plaque accumulation by inhibiting relative gene expression and significantly improve the cognitive performance of mice without increasing the incidence of adverse effects (Yin et al., 2020).

However, the current clinical studies of LSZ lack high-quality evidence and have been limited by methodology, sample size, and non-hard outcome indicators like symptom improvement. Futhermore, the well-documented and transparent statistical analysis is indispensable to reduce publication bias, prevent selective analysis and reporting of research outcomes and support reproducibility, given the influence of statistical decisions on study conclusion. Therefore, we have designed a large-scale, multicenter, double-blind clinical trial to determine the efficacy and safety of the LSZ capsule in patients after AIS (LONGAN). In this paper, we summarized the protocol and statistical analysis plan (SAP) for the primary analysis of LONGAN in accordance with both the Standard Protocol Items: Recommendations for Interventional Trials (SPIRIT) guidelines and the corresponding reporting guideline for SAPs (Chan et al., 2013; Gamble et al., 2017).

## 2 Methods and design

### 2.1 Study design and patient selection

**FIGURE 1:**
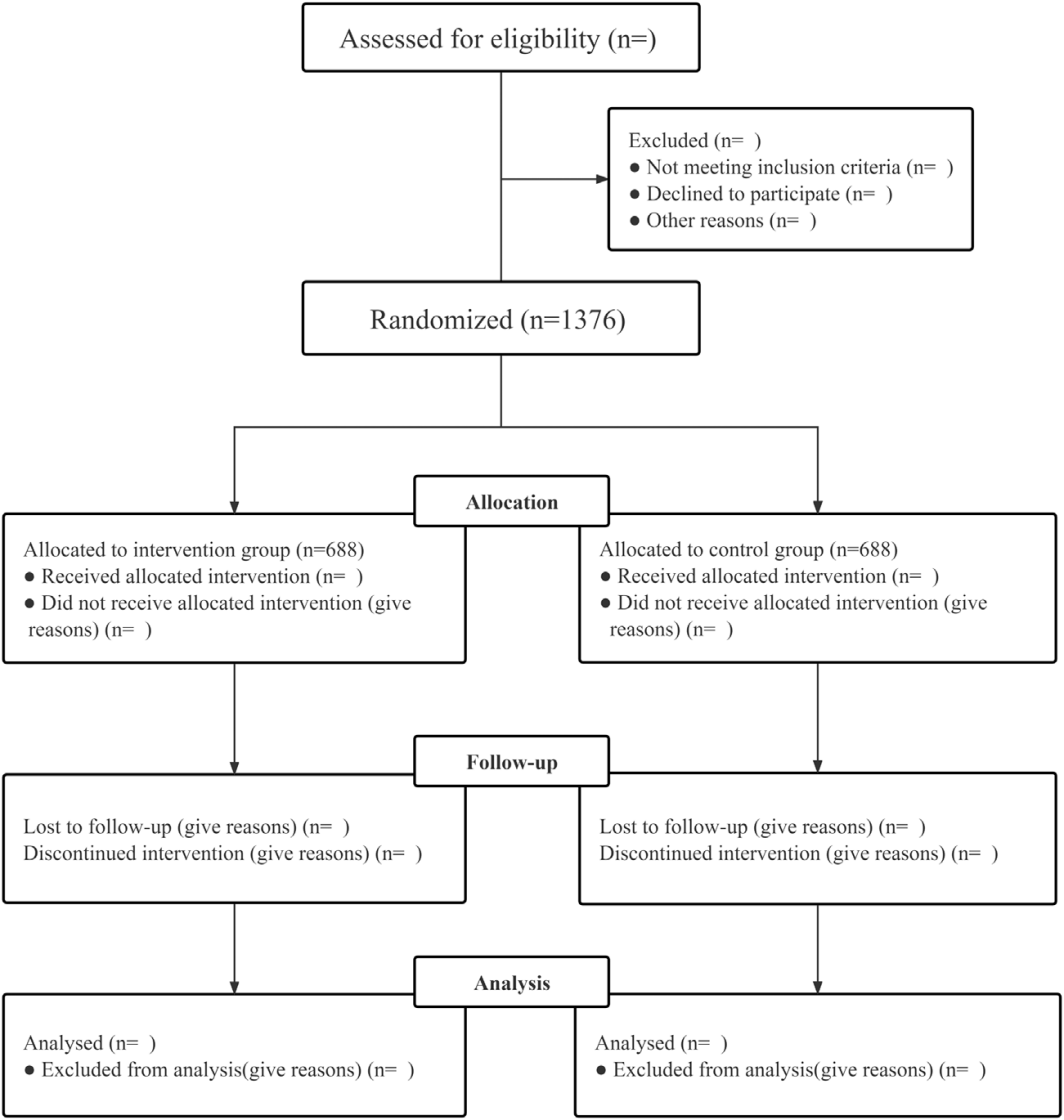
The schematic diagram of the patient timeline.

The LONGAN trial is a prospective, multicenter, randomized, placebo-controlled, double-blind, parallel-group, superiority trial that enrolls patients from stroke and rehabilitation units in China. This study will be conducted at 15 sites across China. The primary objective of the trial is to test the hypothesis that LSZ capsule is superior to the placebo for increasing the proportion of favorable functional status, defined as a modified Rankin Scale (mRS) score ≤1 at 90 days. The secondary objective is the assessment of any other benefits or harms from the end of the treatment period up to 180 days after the stroke. Participants will be followed up at 30, 90 and 180 days after randomization (D30, D90 and D180, respectively) to collect efficacy and safety outcomes in person. Patient enrollment will begin in May 2022 and end in June 2023. Figure 1 presents a schematic diagram of the patient timeline. The trial was approved by the institutional review board of Dongzhimen Hospital, Beijing University of Chinese Medicine, Beijing, China and registered in public clinical trial database (No. 2021DZMEC-198-02; ClinicalTrials.gov Identifier NCT 05277311). We described this study protocol according to the SPIRIT 2013 Statement, and the complete checklist is available.

All patients admitted for a course of inpatient rehabilitation following AIS will be recruited and screened for eligibility based on inclusion and exclusion criteria in the study. Also, the study will be advertised on posters in these medical centers, allowing patients to voluntarily contact investigators. Patients will be further screened for study eligibility for AIS treatment through standard admission assessments: comprehensive medical, neurological, and psychiatric histories; reviews of medical records; physical and neurological examinations; reviews of brain imaging reports and electrocardiogram; and pregnancy test only for the premenopausal women. Furthermore, registration data for eligible patients will be collected. Logs of all patients excluded from the study will be retained to document the reasons for exclusion. Screening logs will be submitted to the coordination center monthly. Eligible patients will be informed of the risks and benefits of the study by trained research clinicians. If they agree to participate in the study, patients and/or their legal surrogates will provide written informed consent forms. The intervention will be initiated as soon as possible after randomization. The detailed diagnostic criteria, inclusion and exclusion criteria are presented in Table 1.

**TABLE 1:** Inclusion and exclusion criteria of the LONGAN trial.

Inclusion criteria
Acute ischemic stroke patients within 7 days of onset
18 years of age or older, and gender not limited
NIHSS score of 4–15 at randomization
**Exclusion criteria**
Secondary stroke caused by a tumor, traumatic brain injury, hematological disease, or other diseases with a confirmed diagnosis
Pre-stroke mRS score of more than 1
Known severe liver or kidney dysfunction
Known allergies for ingredients in the investigational product
Known bleeding diathesis or coagulation disorder
Known medical condition likely to limit survival to less than 3 months
Pregnant women (clinically evident) or breastfeeding women
Participation in any investigational study in the previous 3 months
Known dementia, uncontrolled psychiatric problems
Any condition that could impose hazards to the patient if study therapy is initiated or affect the participation of the patient in the study. The judgment is left to the discretion of the investigator

NIHSS, national institute of health stroke scale; mRS, modified Rankin Scale.

### 2.2 Randomization, allocation, and blinding

Eligible patients who consent to participate will be assigned to the LSZ capsule experimental or control group at a 1:1 ratio using permuted block randomization stratified according to medical centers. The randomization program will be generated by an independent statistician using SAS software v9.4 (SAS Institute, Inc.; Cary, NC, United States) and stored in sealed, sequential, opaque envelopes. The site pharmacist will retain these envelopes, which will be assigned in the order specified by the statistician.

At randomization, study medications will be packaged into a large box containing LSZ capsules or LSZ placebo capsules. When the patient is discharged, the trial medication will be continued and documented on the discharge summary and on the patient’s list of ongoing medications. At 30 days after randomization, patients will be instructed to bring partial empty boxes to this follow-up. Patients who stop taking the allocated treatment early will be followed up, and their data will be included in the primary analyses. The reason for stopping the treatment prematurely, e.g., a SAE, will be recorded in the patient’s electronic case report form (CRF).

Active and placebo capsules with identical appearances, colors, and flavors will be prepared by Buchang Pharmaceutical Co, Ltd. The placebo capsules are visually identical to the LSZ capsules, even when broken open. In the study, each patient’s medications will be prepared as a unit-dose kit according to the predetermined randomization schedule. The site pharmacies will not disclose the randomization assignments unless information is required for patient treatment in exceptional circumstances. The clinical center pharmacist will be unblinded to the treatment, but all investigators, participants, caregivers, and data analysts will be blinded to treatment assignments throughout the trial until the blind codes are revealed. Investigators can request emergency unblinding in cases of serious adverse events (SAEs) suspected to be associated with the investigational medicine.

### 2.3 Treatment

**FIGURE 2:**
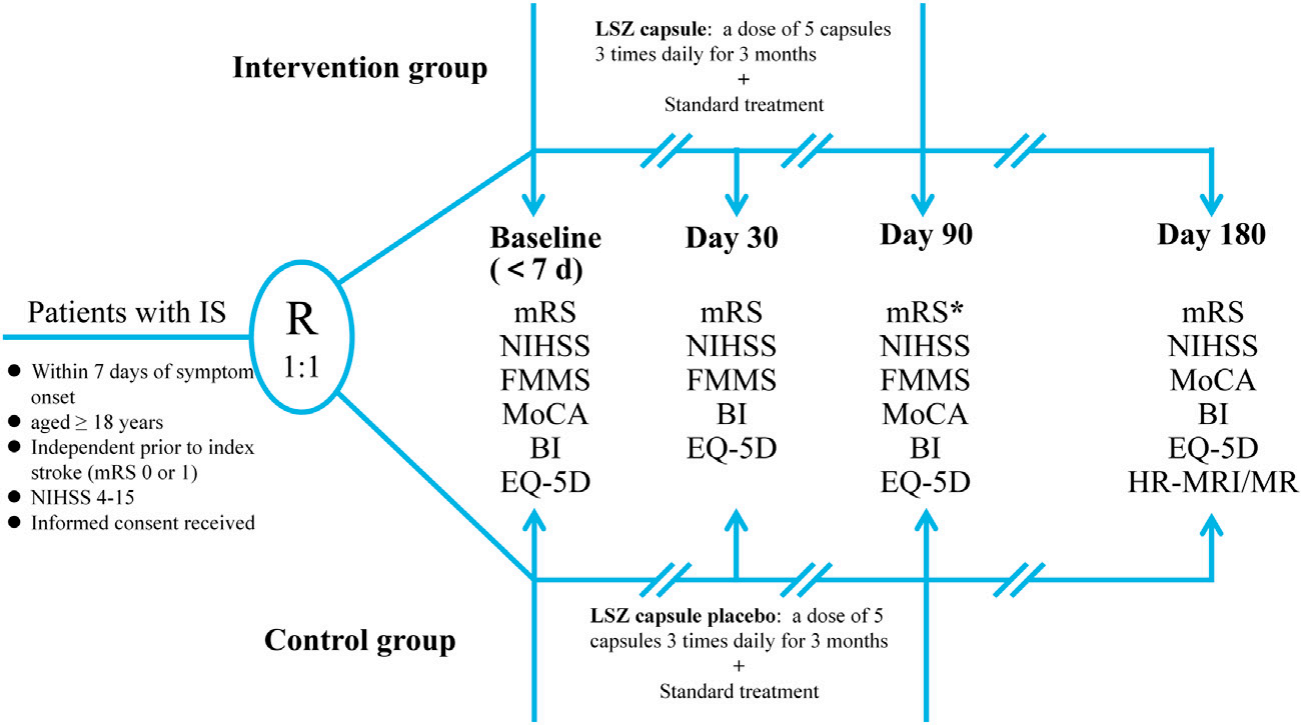
The flowchart of the LONGAN trial. IS, ischemic stroke; mRS, modified Rankin Scale; NIHSS, National Institute of Health Stroke Scale; FMMS, Fugl–Meyer Motor Scale; MoCA, Montreal Cognitive Assessment; BI, Barthel Index; EQ-5D, EuroQol-5D Questionnaire; LSZ, LongShengZhi;HR-MRI, high-resolution magnetic resonance; MR, magnetic resonance.

Eligible patients will be randomly assigned to the experimental and control groups. Participants in the experimental group will receive 5 LSZ capsule (0.4 g each), three times a day for 90 days. The control group will receive a LSZ placebo using the aforementioned protocol; the main ingredients of placebo including starch, dextrins, silicon dioxide, medical yellow iron oxide, caramel color liquid, and purified water will be identical to the LSZ capsule in appearance, weight, and taste. The treatment assignments are presented in Figure 2. All patients will receive a standard treatment, which is referred to in the current guidelines (Chinese Society of Neurology and Chinese Stroke Society, 2018; Powers et al., 2019), including antiplatelet therapy, control of vascular risk factors, and appropriate rehabilitation. During the trial, drugs with composition or efficacy similar to the study drug, such as CM decoctions (compound granules), CM injections, and Chinese patent medicine and health products, will not be permissible. Other concomitant medications will be allowed during the study, but these should be recorded in the subjects’ CRF during the trial. The study drug will be discontinued in cases of SAEs, request for study withdrawal by the patient or their legally authorized representative, or poor compliance or non-adherence to the prescribed interventions. We will truthfully record the reasons for stopping interventions.

### 2.4 Efficacy outcomes

The primary outcome will be the proportion of patients with a favorable outcome, which is defined as mRS ≤1 on D90 (Rankin, 1957). The mRS is an ordinal scale ranging from 0 to 6 (Lees et al., 2012), with 0–1 indicating no disability, 2 to 5 indicating disability (increasing from 2 to 5), and 6 indicating death. The secondary outcomes will be as follows: 1) distribution of mRS scores [will be measured on D30 and D90]; 2) proportion of patients with good functional status [defined as mRS score ≤2; will be measured on D30 and D90]; 3) neurological deficit improvement [defined as a change in the National Institute of Health Stroke Scale (NIHSS) score (Brott et al., 1989), ranging from 0 to 42, with higher scores indicating more severe strokes; will be measured at baseline, D30, and D90]; 4) proportion of poor health-related quality of life (poor-HRQOL) [available EuroQol-5D Questionnaire (EQ-5D) data will be used to calculate EQ-5D index scores (Dorman et al., 1997). Poor-HRQOL is defined as an EQ-5D index score ≤0.5 and will be measured on D30 and D90. The EQ-5D index score is measured on a scale between 0 (death) and 1 (full health). The patient’s mobility, self-care ability, daily activities, feelings of pain or discomfort, and feelings of anxiety or depression contribute to EQ-5D. Here, each aspect will be divided into three levels (Rabin and de Charro, 2001): no problems, moderate problems, or extreme problems. The EQ-5D index score (preference-based health status) will be determined using the population-based preference weight values for five dimensions of the scale (Dolan, 1997; Shaw et al., 2005; Liu et al., 2014). This study will use preference weights applicable to the Chinese population (Liu et al., 2014)]; 5) proportion of patients with functional independence [defined as Barthel Index (BI) ≥90 (Mahoney and Barthel, 1965); BI is a conventional scale used to assess the activities of the daily living of stroke patients involving 10 items with a maximum score of 100 points; will be measured on D30 and D90]; 6) changes in motor function [measured by the Fugl–Meyer Motor Scale (FMMS) score (Fugl-Meyer et al., 1975), ranging from 0 to 100, with lower scores indicating poorer motor status; the change from baseline of the FMMS will be measured on D90]; and 7) changes in cognitive function [measured by the Montreal Cognitive Assessment (MoCA) score (Nasreddine et al., 2005), ranging from 0 to 30, with less than 26 indicating cognitive impairment; the change in MoCA from baseline will be measured on D90].

### 2.5 Exploratory outcomes

In this trial, the exploratory outcomes will be as follows: 1) In the experimental and control groups, 30 cases will be randomly selected from patients who have had previous intracranial artery stenosis in the past, or at their current admission, to compare changes in blood vessel characteristics and plaques under high-resolution magnetic resonance (HR–MR) between the two groups after 180 days; 2) The magnetic resonance imaging (MRI) diffusion tensor imaging (DTI) anisotropy score, average diffusion rate, and diffusion tensor fiber bundle imaging will be compared between the two groups [30 cases will be randomly selected from each group; will be measured on D180]; 3) The proportion of patients with a good function status [defined as mRS score ≤2] will be measured on D180; 4) The distribution of the mRS scores will be measured on D180; 5) Motor function will be assessed by the FMMS score, which will measure its change from baseline at 180 days; 6) Cognitive function will be assessed by the MoCA score; its change from baseline will be measured on D180; 7) Recurrence rate of stroke [defined as cerebral infarction, cerebral hemorrhage] will be measured on D180; 8) The proportion of new combined vascular events [defined as ischemic stroke, hemorrhagic stroke, myocardial infarction, or vascular death] will be measured on D180.

### 2.6 Safety outcomes

The main safety outcome is the proportion of SAEs. Other safety outcomes include any adverse events (AEs) and clinically meaningful changes in vital signs or laboratory parameters during the study period.

### 2.7 Follow-up procedures

The study will consist of four visits, including time of randomization (baseline) and 30, 90, and 180 days after randomization. At baseline, we will evaluate demographic characteristics; routine laboratory test results; computed tomography (CT)/MRI results; electrocardiogram (ECG) results; and NIHSS, Alberta Stroke Program Early CT Score (ASPECTS) (Barber et al., 2000), mRS, FMMS, MoCA, BI and EQ-5D scores. Stroke types will be classified according to the trial of ORG 10172 in acute stroke treatment (TOAST) criteria. At 30, 90, and 180 days after randomization, we will perform assessments using the mRS, NIHSS, FMMS, MoCA, BI and EQ-5D scores. Vital signs and physical examination findings will be recorded at both baseline and 90 days. Finally, medication use for comorbidities and adverse events information will be recorded at whenever they occur during the trial.

### 2.8 Data collection and management

Data collection and management will be conducted in collaboration with clinicians and clinical research coordinators. All researchers responsible for patient recruitment, results evaluation, and data collection will receive standardized training on the standard operating procedures of this trial prior to patient recruitment. During the pilot study, there will be personnel responsible for follow-ups. Patient information will be recorded by the investigator in the patients’ CRF. Subsequently, the clinical research coordinator will use the unique login identity document (ID) to enter the data into the electronic CRF. All patient-related information will be stored in a locked file cabinet in the medical center with limited access rights. All reports, data collection, and administrative forms will be identified by coded ID numbers to protect the confidentiality of participants.

### 2.9 Quality control and data monitoring

The steering committee will be responsible for the scientific content of the protocol, supervision of research operations, the internal data sharing process, and preparation of preliminary manuscripts and other publications produced by the study. Quality control will be applied at every stage of data processing to ensure that all data is reliable and processed correctly. A Data and Safety Monitoring Board (DSMB), whose members are independent of the researchers and steering committee, will periodically evaluate the progress of the clinical trials, safety data, and clinical efficacy endpoints. A mid-term efficacy analysis is planned. After recruiting 50% of patients, a DSMB report should be prepared. If considered necessary, DSMB can request additional reports. If an unexpected safety issue arises or a treatment difference is found during a pre-specified interim analysis, DSMB is responsible for recommending early termination of the study. Two contract research organizations will regularly monitor data and conduct data quality control. Data analysis will be completed by a third-party statistical unit (Beijing Tiantan Hospital, Capital Medical University, Beijing, China). An auditing will be conducted twice a month during the enrollment period and every month during the follow-up period, and the process will be conducted without the involvement of investigators and the sponsor.

### 2.10 Adverse events management

All information about AEs mentioned by the subject, discovered by the investigator, or discovered through physical examination, laboratory examination, or other methods, should be recorded on the adverse event page of the CRF, managed in accordance with corresponding regulations, and reported. Subsequently, the patient will be treated, the treatment outcome will be recorded, and they will be followed up until they recover or stabilize. The investigator will report any SAEs to the ethics committee, contract research agency, chief investigator, and the China Food and Drug Administration.

### 2.11 Sample size calculations and interim analysis

The 90-days mRS distribution of the eligible population in The Third China National Stroke Registry (CNSR-Ⅲ) indicates that the proportion of mRS ≤1 is 60.42% (Wang et al., 2019). This study assumes that the proportion of patients with mRS ≤1 in the control group will be 60%, and the proportion of patients with mRS ≤1 in the experimental group will be increased by approximately 15% for a proportion of 69%. According to this assumption and calculations using PASS 11.0 software (NCSS, LLC, East Kaysville, UT, United States), a total sample size of 1,238 in the two groups can achieve 90% power and rule out two-sided type I errors of 5% to detect a superiority margin difference of 5% in this two-arm trial, with an allocation ratio of 1:1. With an estimated loss-to-follow-up rate of 10%, a total of 1,376 participants will be recruited. An interim analysis will be performed when the 50% of recruited patients (619) complete the study. The interim analysis will allow the DSMB to decide whether to continue or terminate the study. Based on the existing data and the expected final sample size, if this study is not expected to obtain efficacious results, the study will be terminated immediately in the current period. For evaluation of the effectiveness of the research, if the research has met the validity standard and meets the requirements of the inspection level, the study can be terminated early. The study will continue if the estimated efficacy is expected to be achieved by the end of the study. If the estimates of the primary outcome in the study (blinded) differ significantly from the actual values, the final sample size allows for appropriate adjustments at the interim analysis.

### 2.12 Statistical analysis plan

#### 2.12.1 Full analysis set

Statistical analysis will be conducted by a third-party statistical unit using SAS v9.4 software (SAS Institute, Inc.). The full analysis set (FAS) will be the data set as close as possible to the ideal set of subjects in compliance with the intention-to-treat principle, and all subjects who are randomized into the group and have more than one medication record and evaluation of its efficacy will be included in the FAS. Missing data will be imputed using the last observation carried forward (LOCF) method if there are efficacy values after randomization. The FAS will be used for primary efficacy analysis in this study, and all efficacy variables will be analyzed using the FAS.

#### 2.12.2 Per-protocol set

The per-protocol set (PPS) will include all subjects who complete protocol-specified treatments without serious protocol violations. The exact definition of a serious protocol violation will be finalized at the time of data review, which may generally include (but will not be limited to) the following situations: failure to meet the main inclusion criteria, other treatment that seriously interferes with efficacy evaluation after inclusion, poor compliance, and follow up after the time window for follow-up has closed. PPS is a secondary analysis set for validity, but if the results of the FAS and PPS are inconsistent, sensitivity analyses and subgroup analyses will be conducted, and possible causes of the inconsistencies will be investigated.

#### 2.12.3 Safety set

In this trial, the safety analysis will be performed using the safety analysis set (SS). The SS will include those who receive at least one LSZ capsule treatment and one safety evaluation during the study period.

#### 2.12.4 Analysis of the primary outcome

The primary outcome, the proportion of patients with favorable outcome in each group, will be compared by the chi-square test. Assessment of the primary effect will involve the analysis of differences in mRS scores between the LSZ treatment and placebo groups using ordinal logistic regression; the results will be expressed as odds ratios (ORs) with 95% confidence intervals (CIs). Both covariate-adjusted analysis and covariate-unadjusted analysis will be performed. Adjusted analyses will incorporate the following covariates: age, sex, baseline National Institutes of Health Stroke Scale (NIHSS) score, TOAST classification. Adjusting for prognostic factors enhances statistical power in clinical trials, which can also correct for imbalances in baseline prognostic variables, reducing data variability (Optimising the Analysis of Stroke Trials (OAST) Collaboration et al., 2009; Kahan et al., 2014).

#### 2.12.5 Analysis of the secondary and exploratory outcomes

For dichotomous outcomes, including the proportion of patients of good functional status (mRS ≤2), the proportion of poor-HRQOL (EQ-5D index score ≤0.5), the proportion of patients with functional independence (BI ≥ 90), the distribution of mRS scores, recurrence rates of stroke, and the proportion of new combined vascular events, a comparison of how patients with these outcomes are distributed among the two groups will be assessed using a chi-square test or Fisher’s exact test and presented as OR estimates and 95% CIs using logistic regression. Moreover, the distribution of functional outcomes on the mRS in both groups will be expressed in histograms. For other secondary outcomes of continuous variable, the changes from baseline to the endpoint of treatment for the above outcomes, including neurological deficit, motor function and cognitive function will be analyzed using the Student’s t test or Wilcoxon rank-sum test where appropriate. For survival data, the Kaplan-Meier method will be used to estimate the survival rate of each group; a survival curve will be drawn, and the log-rank test will be used to evaluate the curative effect. Cox proportional hazards models will be used to calculate hazard ratios (HR) and 95% CIs between the two treatment regimens. The central effect will be set as a random effect in all models. All statistics will be evaluated using two-sided tests, and *p* < 0.05 will be considered statistically significant.

#### 2.12.6 Analysis of safety outcomes

Based on the SS data set, the method of statistical description will be used, and the differences in the incidence of primary and secondary safety endpoints will be compared between the two groups. For most safety endpoints, HR values between the two treatment regimens will be calculated using Cox proportional hazards models; Poisson regression or negative binomial regression will be used for rare events. AEs and SAEs will be listed separately, and a summary analysis will be performed. Within each treatment group, the number and proportion of AE cases will be aggregated separately by tissue system classification and by items of interest. In addition, all deaths and SAEs will be described in detail using case narratives.

#### 2.12.7 Subgroup analyses

A subgroup analysis of the primary objective will be conducted based on the following patient baseline characteristics: age (<65 and ≥65 years), sex (female and male), medical history, TOAST classification, and NIHSS score.

## 3 Discussion

Stroke, characterized by high morbidity, high mortality, high levels of disability, and high recurrence rates, greatly influences the physical and psychological health of patients and imposes serious burdens on their families and society. Therefore, enhancing the recovery of neurological function is important for improving the prognosis of patients with stroke. It is unfortunate that only few patients with non-minor AIS can benefit from revascularization therapy. Because of this, it is critical to search for therapeutic drugs to improve neurological function during the entire progression of IS.

LSZ capsule is a widely used CM formulation for the treatment of cardiovascular and cerebrovascular diseases in China. Previous studies have reported that the LSZ capsule can inhibit platelet adhesion, thereby reducing thrombosis in atherosclerotic mice by reducing oxidative stress and vascular inflammation in the aorta (Li et al., 2019; Ma et al., 2019). In addition, the LSZ capsule has strong antioxidant effects in patients with heart failure (Yang et al., 2020). A systematic review showed that basic therapy combined with LSZ capsule in AIS treatment could improve neurological deficits and quality of life, but the quality of the evidence was low (Su et al., 2021). The lack of high-quality evidence for LSZ efficacy in the treatment of IS in terms of methodology used, sample size, and non-hard outcome indicators like “symptoms improved” led us design a large-scale, multicenter, double-blind, placebo-controlled clinical trial to furnish higher quality evidence of the safety and efficacy of LSZ capsule treatment for patients with AIS.

MRS is the most prevalent and most often used primary outcome according to a systematic examination of trials involving interventions for stroke from 2007 to 2010 listed on ClinicalTrials.gov (Lees et al., 2012). The mRS is a scale that measures the degree of disability or dependence in the performance of daily activities. In this LONGAN trial, the proportion of patients with favorable outcome, defined as mRS ≤1 at 90 days, has been selected as the primary outcome to assess the therapeutic value of LSZ. Additionally, this paper describes pre-planned analyses for the LONGAN trial, aiming to reduce the risk of data-driven result reporting to support reproducibility and transparency.

To our knowledge, the LONGAN trial is the largest prospective, multicenter, randomized, placebo-controlled, double-blind, parallel-group, superiority trial to assess the efficacy and safety of LSZ capsule in patients with AIS. The study has potential to answer the vexing question of whether LSZ can improve functional recovery in AIS patients. We anticipate LONGAN to be a landmark herbal study and an expected conclusive “end of discussion” report of LSZ. Among that, LONGAN will be conducted on China Stroke Registry for Patients with Traditional Chinese Medicine (CASES-TCM) platform which was the largest nationwide registry (Feng et al., 2021). Participating sites which were required to admit over 100 cases of patients with stroke per year formed a network across the country and offer a wealth of cases. All these strengths and resources will ensure that the LONGAN trial can be completed in a relatively short period of time. The ongoing COVID-19 pandemic has a potentially negative impact on the management of clinical trials. A more flexible approach removing unnecessary barriers may improve enrollment and access to the LONGAN, such as remote monitoring and tele-health visits. Also, this proposed study has two limitations. First, it will not be possible to follow up imaging changes in each patient due to funding constraints. Second, the treatment period will be 90 days and the follow-up period will be 180 days which is relatively short. Due to the limited time frame, the potential roles of LSZ in reducing overall mortality and future major vascular events will be uncertain, and further data on long-term clinical efficacy and safety will be needed. In conclusion, the LONGAN trial will further provide critical and high-quality clinical evidence for the target population of LSZ capsule for patients with mild-to-moderate AIS, regardless of whether the patients have experienced revascularization treatment or not.
