# Supplementary material for: Efficacy and safety of LongShengZhi capsule on functional recovery after acute ischemic stroke (LONGAN): Protocol and statistical analysis plan for a randomized, double-blind, placebo-controlled trial
Source: Front Pharmacol. 2022 Aug 24;13:916421. doi: 10.3389/fphar.2022.916421 (PMC9448855; doi:10.3389/fphar.2022.916421)
Supplement: Supplementary file 1 [file Presentation1.pdf]

Supplementary Files

Appendix 1 Standard formulation of LongShengZhi Capsule

Appendix 2 Standardized Protocol Items for LONGAN

Appendix 1. Standard formulation of LongShengZhi Capsule.

Pinyin name	Complete species name	Authorities / family name	Scientific name	Proportion(g)
Huangqi	Astragalus mongholicus Bunge	Fabaceae	Astmgali Radix	30
Shuizhi	Hirudo nipponica Whitman	Hirudinidae	Hirudo	6
Chuanxiong	Conioselinum anthriscoides 'Chuanxiong'	Apiaceae	Chuanxiong Rhizoma	10
Danggui	Angelica sinensis (Oliv.) Diels	Apiaceae	Angelica sinensis Radix	10
Honghua	Carthamus tinctorius L.	Asteraceae	Carthami Flos	10
Taoren	Prunus persica (L.) BatschChishao	Rosaceae	Persicae Semen	10
Chishao	Paeonia lactiflora Pall. [Paeoniaceae]	Paeoniaceae	Paeoniae Radix Rubra	10
Muxiang	Dolomiaea costus (Falc.) Kasana & A.K.Pandey	Asteraceae	Aucklandiae Radix	6
Shichangpu	Acorus gramineus Aiton	Acoraceae	Acori Tatarinowii Rhizoma	10
Dilong	Pheretima aspergillum (E. Perrier)	Megascolecidae	Pheretima	10
Sangjisheng	Taxillus chinensis (DC.) Danser	Loranthaceae	Talxilli Herba	10

Ciwujia	Eleutherococcus senticosus (Rupr. & Maxim.) Maxim.	Araliaceae	Acanthopanax senticosus	10

Appendix 2 Standardized Protocol Items for LONGAN

Section/item	ltem No.	Description	Addressed on page number
**Administrative information**			
Title	1	Efficacy and Safety of LongShengZhi Capsule on Functional Recovery after Acute Ischemic Stroke (LONGAN): Protocol and Statistical Analysis Plan for a Randomized, Double-Blind, Placebo-Controlled Trial	Page 1, line 1
Trial registration	2a	ClinicalTrials.gov: NCT 05277311	Page 5, line 129
Protocol version	3	12 Jan 2022	N/A
Funding	4	This trial was supported by grants from the Chinese Medicine Inheritance and Innovation Talent Project-National Leading Talent Support Program for Traditional Chinese Medicine 2018 (No.12), Special Subjects in Fundamental Scientific and Research Expenses based Project of Beijing University of Chinese Medicine (No. 2020-JYB-TSXK-001), the Dongzhimen Hospital Project (2020TSRC-002), and Buchang Pharmaceutical Co., Ltd.	Page 13, line 456
**Introduction**			
Background and rationale	6a	Stroke is a common disease affecting a quarter of the world's population and is the leading cause of death and disability. Ischemic stroke (IS), accounting for 70% of strokes, is the most prevalent type of stroke and occurs mainly due to decreased cerebral blood flow caused by thrombosis or embolism. Current treatments for acute ischemic stroke (AIS) include reperfusion and antiplatelet therapy and admission to the stroke unit. Unfortunately, only a small proportion of patients can undergo reperfusion treatment due to the limited time window and technical requirements. Improvement of the neurological function of patients with AIS remains the main focus and is a difficult issue in clinical practice. During the past two decades, many neuroprotective agents have shown promising results in animal experiments, but it is inconclusive whether they can be translated to clinical trials. To date, there are no guideline-approved pharmacotherapies for neuroprotection after AIS. Promotion of nerve function recovery remains one of the most important therapeutic strategies for AIS; thus, more appropriate and effective medication options are urgently needed for the improvement of neurological function after AIS.<br><br>Chinese medicine (CM), a unique and complicated medical system, has been developed over thousands of years and is widely applied as a supplementary and complementary therapy to Western medicine in IS treatment.The LongShengZhi (LSZ) capsule, a well-known traditional Chinese herbal medicine product for treating IS, is formulated through the modification of a representative prescription (Buyang Huanwu decoction) proposed by Wang Qing-ren during the Qing dynasty. Moreover, other studies have indicated that the LSZ capsule has anti-inflammatory and neuroprotective effects and can reduce the activation of platelets and endothelial cells. Based on a	Page 4, line 60

		network pharmacology analysis, the LSZ capsule can regulate the pathological process of IS through regulation of inflammation processes, trophic factor secretion, and immune-related lymphocyte regulation.<br><br>However, the current clinical studies of LSZ lack high-quality evidence and have been limited by methodology, sample size, and non-hard outcome indicators like symptom improvement. Moreover, given the influence of statistical decisions on study conclusions, well-documented and transparent statistical analysis is indispensable to reduce publication bias and prevent selective analysis and reporting of research outcomes, as well as to support reproducibility. Therefore, we have designed a large-scale, multicenter, double-blind clinical trial to determine the efficacy and safety of the LSZ capsule in patients after AIS (LONGAN), to assess the efficacy and safety of LSZ in patients with IS. In this paper, we summarized the protocol and statistical analysis plan (SAP) for the primary analysis of LONGAN in accordance with both the Standard Protocol Items: Recommendations for Interventional Trials (SPIRIT) guidelines and the corresponding reporting guideline for SAPs.	
	6b	The control group will receive a LSZ placebo using the aforementioned protocol; the main ingredients of starch, dextrins, silicon dioxide, medical yellow iron oxide, caramel color liquid, and purified water will be identical to the LSZ capsule in appearance, weight, and taste.Active and placebo capsules with identical appearances, colors, and flavors will be prepared by Buchang Pharmaceutical Co, Ltd. All patients will receive a standard treatment, which is referred to in the current guidelines, including antiplatelet therapy, control of vascular risk factors, and appropriate rehabilitation. During the trial, drugs with composition or efficacy similar to the study drug, such as CM decoctions (compound granules), CM injections, and Chinese patent medicine and health products, will not be permissible.	Page 6, line 187
Objectives	7	The trial aims to provide high-quality evidence for the efficacy and safety of LSZ capsule for AIS.	Page 3, line 47
Trial design	8	The LONGAN trial is a prospective, multicenter, randomized, placebo-controlled, double-blind, parallel-group, superiority trial that enrolls patients from stroke and rehabilitation units in China. Eligible patients who consent to participate will be assigned to the LSZ capsule experimental or control group at a 1:1 ratio using permuted block randomization stratified according to medical centers.	Page 5, line 118
**Methods: Participants, interventions, and outcomes**			
Study setting	9	This study will be conducted at 15 sites across China.	Page 5, line 119
Eligibility criteria	10	**Inclusion criteria**:<br>1) Acute ischemic stroke patients within 7 days of onset;<br>2) 18 years of age or older, and gender not limited;<br>3) NIHSS score of 4-15 at randomization.<br>**Exclusion criteria**:	Page 21, line 602

		➢ Secondary stroke caused by a tumor, traumatic brain injury, hematological disease, or other diseases with a confirmed diagnosis;<br>➢ Pre-stroke mRS score of more than 1;<br>➢ Known severe liver or kidney dysfunction;<br>➢ Known allergies for ingredients in the investigational product;<br>➢ Known bleeding diathesis or coagulation disorder;<br>➢ Known medical condition likely to limit survival to less than 3 months;<br>➢ Pregnant women (clinically evident) or breastfeeding women;<br>➢ Participation in any investigational study in the previous 3 months;<br>➢ Known dementia, uncontrolled psychiatric problems;<br>➢ Any condition that could impose hazards to the patient if study therapy is initiated or affect the participation of the patient in the study. The judgment is left to the discretion of the investigator.	
Interventions	11a	Participants in the experimental group will receive 5 LSZ capsule (0.4 g each), three times a day for 90 days. The control group will receive a LSZ placebo using the aforementioned protocol;	Page 6, line 185
	11b	If the followings occur during the trial, the subjects will withdraw from the trial.<br>The investigators decided to withdraw:<br>(1)The subjects are aggravated and unable to continue the trial.<br>(2)Anaphylaxis or serious adverse events should be stopped according to the doctor's judgment.<br>(3)If the subjects are found to meet the exclusion criteria after randomization, withdraw the trial medication from the subjects.<br>(4)Subjects have poor compliance with medication and are not standardized.<br>(5)Other reasons researchers believe subjects should withdraw from the trial.<br>The subjects decided to withdraw:<br>(1)For whatever reason, the subject is unwilling or unlikely to continue the trial, asking the researcher to withdraw from the study and suspending the researcher.<br>(2)Although the subjects do not explicitly withdraw from the study, they no longer accept medication and testing and are lost to follow-up.	
	11c	At randomization, study medications will be packaged into a large box containing LSZ capsules or LSZ placebo capsules. When the patient is discharged, the trial medication will be continued and documented on the discharge summary and on the patient’s list of ongoing medications. At 30 days after randomization, patients will be instructed to bring partial empty boxes to this follow-up.	Page 6, line 168
	11d	During the trial, drugs with composition or efficacy similar to the study drug, such as CM decoctions (compound granules), CM injections, and Chinese patent medicine and health products, will not be permissible. Other concomitant medications will be allowed during the study, but these should be recorded in the subjects’ CRF	Page 7, line 193

		during the trial.	
Outcomes	12	**Efficacy Outcomes**<br>The primary outcome will be the proportion of patients with a favorable outcome, which is defined as mRS ≤1 on D90. The secondary outcomes will be as follows: 1) distribution of mRS scores [will be measured on D30 and D90]; 2) proportion of patients with good functional status [defined as mRS score ≤2; will be measured on D30 and D90]; 3) neurological deficit improvement [defined as a change in the National Institute of Health Stroke Scale (NIHSS) score; will be measured at baseline, D30, and D90]; 4) proportion of poor health-related quality of life (poor-HRQOL) [available EuroQol-5D Questionnaire (EQ-5D) data will be used to calculate EQ-5D index scores. Poor-HRQOL is defined as an EQ-5D index score ≤0.5 and will be measured on D30 and D90; 5) proportion of patients with functional independence [defined as Barthel Index (BI) ≥90,measured on D30 and D90]; 6) changes in motor function [measured by the Fugl–Meyer Motor Scale (FMMS) score, the change from baseline of the FMMS will be measured on D90]; and 7) changes in cognitive function [measured by the Montreal Cognitive Assessment (MoCA) score, the change in MoCA from baseline will be measured on D90].<br>**Exploratory Outcomes**<br>In this trial, the exploratory outcomes will be as follows. 1) In the experimental and control groups, 30 cases will be randomly selected from patients who have had previous intracranial artery stenosis in the past, or at their current admission, to compare changes in blood vessel characteristics and plaques under high-resolution magnetic resonance (HR–MR) between the two groups after 180 days. 2) The magnetic resonance imaging (MRI) diffusion tensor imaging (DTI) anisotropy score, average diffusion rate, and diffusion tensor fiber bundle imaging will be compared between the two groups [30 cases will be randomly selected from each group; will be measured on D180]. 3) The proportion of patients with a good function status [defined as mRS score ≤2] will be measured on D180. 4) The distribution of the mRS scores will be measured on D180. 5) Motor function will be assessed by the FMMS score, which will measure its change from baseline at 180 days. 6) Cognitive function will be assessed by the MoCA score; its change from baseline will be measured on D180. 7) Recurrence rate of stroke [defined as cerebral infarction, cerebral hemorrhage] will be measured on D180. 8) The proportion of new combined vascular events [defined as ischemic stroke, hemorrhagic stroke, myocardial infarction, or vascular death] will be measured on D180.<br>**Safety Outcomes**<br>The main safety outcome is the proportion of SAEs. Other safety outcomes include any adverse events (AEs) and clinically meaningful changes in vital signs or laboratory parameters during the study period.	Page 7, line 201
Participant	13	Please see Figure2.	Page 19, line 593

timeline			
Sample size	14	The 90-day mRS distribution of the eligible population in The Third China National Stroke Registry indicates that the proportion of mRS ≤1 is 60.42%(Wang et al., 2019). This study assumes that the proportion of patients with mRS ≤1 in the control group will be 60%, and the proportion of patients with mRS ≤1 in the experimental group will be increased by approximately 15% for a proportion of 69%. According to this assumption and calculations using PASS 11.0 software (NCSS, LLC, East Kaysville, UT, USA), a total sample size of 1238 in the two groups can achieve 90% power and rule out two-sided type I errors of 5% to detect a superiority margin difference of 5% in this two-arm trial, with an allocation ratio of 1:1. With an estimated loss-to-follow-up rate of 10%, a total of 1376 participants will be recruited.	Page 9, line 294
Recruitment	15	All patients admitted for a course of inpatient rehabilitation following AIS will be recruited and screened for eligibility based on inclusion and exclusion criteria in the study. Also, the study will be advertised on posters in these medical centers to allow patients to voluntarily contact investigators.	Page 5, line 132
**Methods: Assignment of interventions (for controlled trials)**			
**Allocation:**			
Sequence generation	16a	Eligible patients who consent to participate will be assigned to the LSZ capsule experimental or control group at a 1:1 ratio using permuted block randomization stratified according to medical centers. The randomization program will be generated by an independent statistician using SAS software v9.4 (SAS Institute, Inc.; Cary, NC, USA) and stored in sealed, sequential, opaque envelopes.	Page 6, line 162
Allocation concealment mechanism	16b	The randomization program will be generated by an independent statistician using SAS software v9.4 (SAS Institute, Inc.; Cary, NC, USA) and stored in sealed, sequential, opaque envelopes. The site pharmacist will retain these envelopes, which will be assigned in the order specified by the statistician.	Page 6, line 164
Implementation	16c	Eligible patients who consent to participate will be assigned to the LSZ capsule experimental or control group at a 1:1 ratio using permuted block randomization stratified according to medical centers. The randomization program will be generated by an independent statistician using SAS software v9.4 (SAS Institute, Inc.; Cary, NC, USA) and stored in sealed, sequential, opaque envelopes. The site pharmacist will retain these envelopes, which will be assigned in the order specified by the statistician.	Page 6, line 162
Blinding (masking)	17a	The clinical center pharmacist will be unblinded to the treatment, but all investigators, participants, caregivers, and data analysts will be blinded to treatment assignments throughout the trial until the blind codes are revealed. Investigators can request emergency unblinding in cases of serious adverse events (SAEs) suspected to be associated with the investigational medicine.	Page 6, line 180
	17b	In the study, each patient’s medications will be prepared as a unit-dose kit according to the predetermined randomization schedule.	Page 6, line 177

		The site pharmacies will not disclose the randomization assignments unless information is required for patient treatment in exceptional circumstances. The clinical center pharmacist will be unblinded to the treatment, but all investigators, participants, caregivers, and data analysts will be blinded to treatment assignments throughout the trial until the blind codes are revealed. Investigators can request emergency unblinding in cases of serious adverse events (SAEs) suspected to be associated with the investigational medicine.	
**Methods: Data collection, management, and analysis**			
Data collection methods	18a	Data collection and management will be conducted in collaboration with clinicians and clinical research coordinators. All researchers responsible for patient recruitment, results evaluation, and data collection will receive standardized training on the standard operating procedures of this trial prior to patient recruitment. During the pilot study, there will be personnel responsible for follow-ups. Patient information will be recorded by the investigator in the patients' CRF. Subsequently, the clinical research coordinator will use the unique login identity document (ID) to enter the data into the electronic CRF. All patient-related information will be stored in a locked file cabinet in the medical center with limited access rights. All reports, data collection, and administrative forms will be identified by coded ID numbers to protect the confidentiality of participants.	Page 8, line 260
	18b	When the patient is discharged, the trial medication will be continued and documented on the discharge summary and on the patient's list of ongoing medications. At 30 days after randomization, patients will be instructed to bring partial empty boxes to this follow-up. Patients who stop taking the allocated treatment early will be followed up, and their data will be included in the primary analyses. The reason for stopping the treatment prematurely, e.g., a SAE, will be recorded in the patient's electronic case report form (CRF).	Page 6, line 169
Data management	19	Data collection and management will be conducted in collaboration with clinicians and clinical research coordinators. All researchers responsible for patient recruitment, results evaluation, and data collection will receive standardized training on the standard operating procedures of this trial prior to patient recruitment. During the pilot study, there will be personnel responsible for follow-ups. Patient information will be recorded by the investigator in the patients' CRF. Subsequently, the clinical research coordinator will use the unique login identity document (ID) to enter the data into the electronic CRF. All patient-related information will be stored in a locked file cabinet in the medical center with limited access rights. All reports, data collection, and administrative forms will be identified by coded ID numbers to protect the confidentiality of participants.	Page 8, line 260

Statistical methods	20a	**Analysis of the Primary Outcome:**<br>The primary outcome, the proportion of patients with favorable outcome in each group, will be compared by the chi-square test. Assessment of the primary effect will involve the analysis of differences in mRS scores between the LSZ treatment and placebo groups using ordinal logistic regression; the results will be expressed as odds ratios (ORs) with 95% confidence intervals (CIs). Both covariate-adjusted analysis and covariate-unadjusted analysis will be performed. Adjusted analyses will incorporate the following covariates: age, sex, baseline National Institutes of Health Stroke Scale (NIHSS) score, TOAST classification. Adjusting for prognostic factors enhances statistical power in clinical trials, which can also correct for imbalances in baseline prognostic variables, reducing data variability (Optimizing the Analysis of Stroke Trials (OAST) .<br>**Analysis of the Secondary and Exploratory Outcomes:**<br>For dichotomous outcomes, including the proportion of patients of good functional status (mRS ≤2), the proportion of poor-HRQOL (EQ-5D index score ≤0.5), the proportion of patients with functional independence (BI ≥90), the distribution of mRS scores, recurrence rates of stroke, and the proportion of new combined vascular events, a comparison of how patients with these outcomes are distributed among the two groups will be assessed using a chi-square test or Fisher's exact test and presented as OR estimates and 95% CIs using logistic regression. Moreover, the distribution of functional outcomes on the mRS in both groups will be expressed in histograms. For other secondary outcomes of continuous variable, the changes from baseline to the endpoint of treatment for the above outcomes, including neurological deficit, motor function and cognitive function will be analyzed using the Student's t test or Wilcoxon rank-sum test where appropriate. For survival data, the Kaplan–Meier method will be used to estimate the survival rate of each group; a survival curve will be drawn, and the log-rank test will be used to evaluate the curative effect. Cox proportional hazards models will be used to calculate hazard ratios (HR) and 95% CIs between the two treatment regimens. The central effect will be set as a random effect in all models. All statistics will be evaluated using two-sided tests, and $P<0.05$ will be considered statistically significant.<br>**Analysis of Safety Outcomes:**<br>Based on the SS data set, the method of statistical description will be used, and the differences in the incidence of primary and secondary safety endpoints will be compared between the two groups. For most safety endpoints, HR values between the two treatment regimens will be calculated using Cox proportional hazards models; Poisson regression or negative binomial regression will be used for rare events. AEs and SAEs will be listed separately, and a summary analysis will be performed. Within each treatment group, the number and proportion of AE cases will be aggregated separately by tissue system classification and by items of interest. In addition, all deaths and SAEs will be described in detail using case narratives.	Page 11, line 334

	20b	A subgroup analysis of the primary objective will be conducted based on the following patient baseline characteristics: age (≤65 and ≥65 years), sex (female and male), disease history, TOAST classification, and NIHSS score.	Page 12, line 371
	20c	Missing data will be imputed using the last observation carried forward (LOCF) method if there are efficacy values after randomization.	Page 10, line 318
**Methods: Monitoring**			
Data monitoring	21a	The steering committee will be responsible for the scientific content of the protocol, supervision of research operations, the internal data sharing process, and preparation of preliminary manuscripts and other publications produced by the study. Quality control will be applied at every stage of data processing to ensure that all data is reliable and processed correctly. A Data and Safety Monitoring Board (DSMB), whose members are independent of the researchers and steering committee, will periodically evaluate the progress of the clinical trials, safety data, and clinical efficacy endpoints.	Page 9, line 270
	21b	An interim analysis will be performed when the 50% of recruited patients (619) complete the study. The interim analysis will allow the DSMB to decide whether to continue or terminate the study. Based on the existing data and the expected final sample size, if this study is not expected to obtain efficacious results, the study will be terminated immediately in the current period. For evaluation of the effectiveness of the research, if the research has met the validity standard and meets the requirements of the inspection level, the study can be terminated early. The study will continue if the estimated efficacy is expected to be achieved by the end of the study. If the estimates of the primary outcome in the study (blinded) differ significantly from the actual values, the final sample size allows for appropriate adjustments at the interim analysis.	Page 10, line 302
Harms	22	All information about AEs mentioned by the subject, discovered by the investigator, or discovered through physical examination, laboratory examination, or other methods, should be recorded on the adverse event page of the CRF, managed in accordance with corresponding regulations, and reported. Subsequently, the patient will be treated, the treatment outcome will be recorded, and they will be followed up until they recover or stabilize. The investigator will report any SAEs to the ethics committee, contract research agency, chief investigator, and the China Food and Drug Administration.	Page 9, line 286
Auditing	23	An auditing will be conducted twice a month during the enrollment period and every month during the follow-up period, and the process will be conducted without the involvement of investigators and the sponsor.	Page 9, line 281
**Ethics and dissemination**			
Research ethics approval	24	The trial involving human participants were reviewed and approved by the Dongzhimen Hospital, Beijing University of Chinese Medicine (No.2021DZMEC-198). The patients/participants provided their written informed consent to participate in this trial.	Page 13, line 434

Protocol amendments	25	Any modifications to the protocol which may impact on the conduct of the study, patients'potential benefit or safety will require a formal amendment to the protocol. Such amendment will be agreed upon by sponsor and principal investigator, and approved by the Ethics Committee prior to implementation. Version control will use protocol identifiers and dates, as well as list of amendments, to help track the history of amendments and identify the most recent protocol version.	
Consent or assent	26a	Eligible patients will be informed of the risks and benefits of the study by trained research clinicians. If they agree to participate in the study, patients and/or their legal surrogates will provide written informed consent forms.	Page 6, line 156
	26b	N/A	
Confidentiality	27	All patient-related information will be stored in a locked file cabinet in the medical center with limited access rights. All reports, data collection, and administrative forms will be identified by coded ID numbers to protect the confidentiality of participants.	Page 9, line 266
Declaration of interests	28	This study received partial funding from commercial entity of Buchang Pharmaceutical Co., Ltd. The funder was not involved in the study design, collection, analysis, interpretation of data, the writing of this article or the decision to submit it for publication. All authors declare no other competing interests.	Page 13, line 438
